# Using deep learning to detect diabetic retinopathy on handheld non-mydriatic retinal images acquired by field workers in community settings

**DOI:** 10.1038/s41598-023-28347-z

**Published:** 2023-01-25

**Authors:** Joan M. Nunez do Rio, Paul Nderitu, Rajiv Raman, Ramachandran Rajalakshmi, Ramasamy Kim, Padmaja K. Rani, Sobha Sivaprasad, Christos Bergeles, for the SMART India Study Group, Rajiv Raman, Pramod Bhende, Janani Surya, Lingam Gopal, Radha Ramakrishnan, Rupak Roy, Supita Das, George Manayath, T. P. Vignesh, Giridhar Anantharaman, Mahesh Gopalakrishnan, Sundaram Natarajan, Radhika Krishnan, Sheena Liz Mani, Manisha Agarwal, Umesh Behera, Harsha Bhattacharjee, Manabjyoti Barman, Alok Sen, Moneesh Saxena, Asim K. Sil, Subhratanu Chakabarty, Thomas Cherian, Reesha Jitesh, Rushikesh Naigaonkar, Abishek Desai, Sucheta Kulkarni

**Affiliations:** 1 Institute of Ophthalmology, University College London, 11-43 Bath St., London, EC1V 9EL UK; 2 Section of Ophthalmology, King’s College London, London, WC2R 2LS UK; 3 Vision Research Foundation, Chennai, India; 4 Dr. Mohan’s Diabetes Specialities Centre and Madras Diabetes Research Foundation, Chennai, India; 5 Aravind Eye Hospital, Madurai, India; 6 Anand Bajaj Retina Institute, Srimati Kannuri Santhamma Centre for Vitreoretinal Diseases, LV Prasad Eye Institute, Hyderabad, Telangana India; 7 NIHR Moorfields Biomedical Research Centre, Moorfields Eye Hospital, London, UK; 8 School of Biomedical Engineering & Imaging Sciences, King’s College London, London, SE1 7EU UK; 9 Sankara Nethralaya, Chennai, Tamil Nadu India; 10 Vision Sciences, UCL, London, UK; 11 SankaraNethralaya, Kolkata, India; 12 Aravind Eye Hospital, Coimbatore, Tamil Nadu India; 13 Giridhar Eye Institute, Cochin, Kerala India; 14 Aditya Jyot Hospital, Mumbai, Maharashtra India; 15 Dr Tony Fernandez Eye Hospital, Aluva, Kerala India; 16 Dr Shroff’s Charity Eye Hospital, New Delhi, India; 17 LV Prasad Eye Institute, Bhubaneshwar, Odisha India; 18 Sri Sankaradeva Nethralaya, Guwahati, Assam India; 19 Sadguru Netra Chikitsalaya, Chitrakoot, Madhya Pradesh India; 20 Aurobindo Nethralaya, Raipur, Chhattisgarh India; 21 Netra Niramay Niketan, Haldia, West Bengal India; 22 Little Flower Hospital and Research Centre, Angamaly, Kerala India; 23 HV Desai Hospital, Pune, Maharashtra India

**Keywords:** Machine learning, Predictive medicine, Medical imaging

## Abstract

Diabetic retinopathy (DR) at risk of vision loss (referable DR) needs to be identified by retinal screening and referred to an ophthalmologist. Existing automated algorithms have mostly been developed from images acquired with high cost mydriatic retinal cameras and cannot be applied in the settings used in most low- and middle-income countries. In this prospective multicentre study, we developed a deep learning system (DLS) that detects referable DR from retinal images acquired using handheld non-mydriatic fundus camera by non-technical field workers in 20 sites across India. Macula-centred and optic-disc-centred images from 16,247 eyes (9778 participants) were used to train and cross-validate the DLS and risk factor based logistic regression models. The DLS achieved an AUROC of 0.99 (1000 times bootstrapped 95% CI 0.98–0.99) using two-field retinal images, with 93.86 (91.34–96.08) sensitivity and 96.00 (94.68–98.09) specificity at the Youden’s index operational point. With single field inputs, the DLS reached AUROC of 0.98 (0.98–0.98) for the macula field and 0.96 (0.95–0.98) for the optic-disc field. Intergrader performance was 90.01 (88.95–91.01) sensitivity and 96.09 (95.72–96.42) specificity. The image based DLS outperformed all risk factor-based models. This DLS demonstrated a clinically acceptable performance for the identification of referable DR despite challenging image capture conditions.

## Introduction

Diabetic retinopathy (DR) is a common microvascular complication of diabetes^1,2^. Approximately 10% of people with diabetes can progress to vision threatening diabetic retinopathy (VTDR) without any symptoms, whose early signs can include blurred vision, eye pain or redness and the appearance of floating shapes in the vision field^3^. Known risk factors of DR are duration of diabetes, uncontrolled diabetes and hypertension. However, identifying people with VTDR based on models on these risk factors is not accurate. Therefore, it is recommended that every person with diabetes undergoes retinal screening regularly to identify intraretinal signs of DR (microaneurysms, haemorrhage, drusen) and refer those at risk of VTDR (referable DR) for regular monitoring by an ophthalmologist and VTDR treatment when indicated^4,5^. There are approximately 537 million people with diabetes worldwide, and 75% reside in low- and middle-income countries (LMIC)^2^. Approximately 40 million people are at risk of having VTDR in these countries^2^, but establishing systematic DR screening programmes to the standards recommended in high income countries is not feasible in LMIC due to the costs of the retinal cameras (fixed table-top fixed retinal cameras), lack of infrastructure and trained workforce to obtain and grade retinal images.

To ensure screening of large number of people with diabetes and to reach remote and rural areas, most LMIC employ non-technical staff to screen people with diabetes in community settings using non-mydriatic low-cost cameras^2,6^. These screening strategies have additional approach-specific challenges^6,7^. Handheld non-mydriatic retinal cameras offer the benefits of portability and low-cost but they increase the rate of ungradable images, in part due to the lack of a stabilising platform^8^. Image quality is also impacted by the increased prevalence of undiagnosed co-pathology in communities with limited healthcare access, particularly cataract, the most common cause of visual impairment in LMIC^9^.

The recommended workforce for grading retinal images is not cost-effective even in high income countries^10^. One solution to a more efficient and sustainable programme is to employ automated algorithms. Deep learning, as a state-of-the-art machine learning technique, has achieved remarkable success in the detection of a variety of medical conditions, particularly in ophthalmology^11–13^, and most notably DR^14–18^. However, to date, automated algorithms for DR screening have been developed using retinal images acquired through dilated pupils on fixed desktop cameras by a trained workforce^14,15,17,18^. These algorithms cannot be translated to non-mydriatic retinal images captured by field workers in the challenging acquisition conditions of community settings^19^. A substantial proportion of retinal images captured in such environments exhibit variable quality due to obscuration of fundal areas, variable image brightness and suboptimal focus. Therefore, automated algorithms need to be developed specifically for this setting. As such, there is an unmet need for an automated algorithm that grades retinal images taken in non-clinical, community environments to enable the translation and adoption of DR screening in LMIC.

As part of the SMART India study, a cross-sectional study conducted across 20 regions in India, in this work-package we developed and evaluated a deep learning-based system (DLS) in detecting referable DR. We focussed not only on traditional two-field images but also on single-field macula or optic disc-centred handheld non-mydriatic retinal images to inform the accuracy of the algorithm based on the retinal area captured in such settings. In addition, we compared the accuracy of this algorithm to risk-models based on systemic risk factors that are used to identify DR in settings where retinal screening is not available.

## Methods

### Study design and participants

Participants were recruited and screened in two stages between 20th December 2018 and 20th March 2020 (SMART-INDIA 1, SM1) and between 8th October 2020 and 17th April 2021 (SMART INDIA 2, SM2). A stratified sample of adults aged 40 years or above were screened in each household for diabetes, and those with diabetes were screened for DR by minimally trained field workers using low cost handheld non-mydriatic retinal cameras (see included centres in Supplementary Fig. S1)^20^. Field workers underwent on-site training at each centre on the use of a handheld Zeiss Visuscout 100 camera (Zeiss, Germany) to capture a set of at least two 40° colour retinal photographs (macula and optic disc centred) from each eye without pupil dilation. To maximize gradeability rates, no limit was set on the number of acquired photographs for each patient. When difficulties, media opacities or undiagnosed co-pathologies, such as cataract or small pupils, hindered the acquisition of fundus images, photographs of the anterior segment were acquired with the same camera, which were not used in the development of the DLS for referable DR screening. In SM1, field workers captured the set of retinal fundus photographs in community screenings from individuals who had confirmed diabetes or who, on the day of survey, had an elevated random blood sugar of 8.9 mmol/L or higher. In SM2, to enrich the total dataset with VTDR images, the same field workers screened in the ophthalmology clinics only patients who had confirmed diabetes, resulting in a higher prevalence of referable patients.

This cross-sectional study complied with the Declaration of Helsinki and was approved by The Indian Council of Medical Research (ICMR)/Health Ministry Screening Committee (HMSC/2018-0494, dated 17/12/2018). Institutional Ethics Committees of all the participating institutions approved both parts of the study (SM1 and SM2). Informed consent was obtained from each participant. The study protocol has been published^20^.

### Image grading

**Figure 1:**
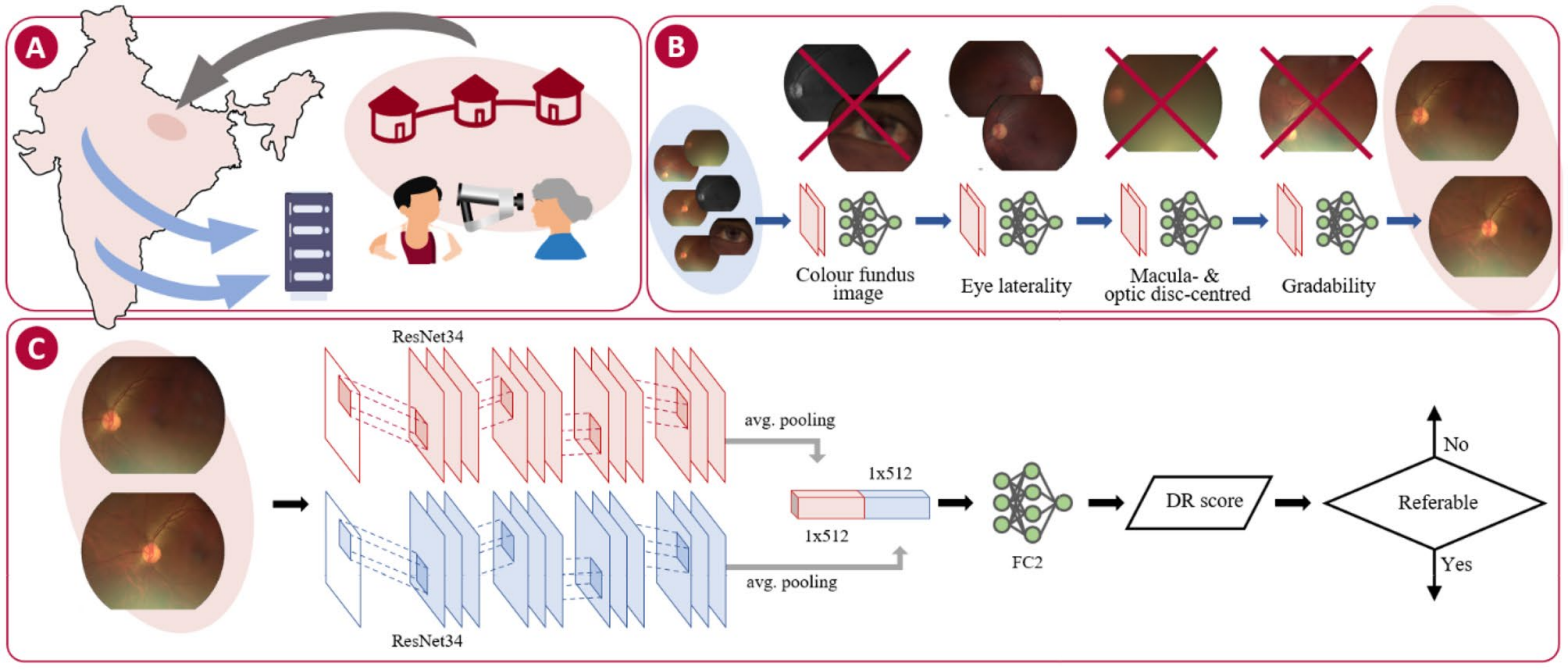
(**A**) Handheld non-mydriatic fundus images are captured by field workers in a community-based study across 20 sites in India. Images are uploaded to a cloud-based central database for independent manual grading at the study site (black arrow) and double graded and arbitrated at the four reading centres (blue arrows). (**B**) An automated pipeline comprising four independent deep learning models is used to curate the retinal for DR grading: fundus image detection, eye laterality detection, gradeability scoring, and field detection. (**C**) Model for automated referable DR grading of retinal photographs: two ResNet34^24^ encoders independently trained extract features from each retinal field followed by global average pooling. The resulting feature maps were concatenated (1 × 1024) and followed by a fully-connected layer for classification (FC2). (Adobe Photoshop CS6 v13.0.1 http://www.adobe.com).

A teleophthalmology system was set up whereby retinal images captured by each fieldworker were uploaded to a cloud-based database for subsequent independent grading at the local clinical centre (on-site primary grading), as well as transferred to four central reading centres for secondary grading (Fig. 1A). Trained optometrists or ophthalmologists graded all images from each eye and discrepancies between primary and secondary grading were arbitrated by a senior retinal consultant at each Reading Centre. Person eyes were classified as per the International Clinical Disease Severity Scale for DR as no DR, mild, moderate, severe non-proliferative DR, and proliferative DR^21,22^, or as ungradable. Gradable eyes had two outcomes: (1) referable DR (moderate non-proliferative DR or worse) or non-referable DR (eyes with no DR or mild DR), and (2) diabetic macular edema (DME) graded as non-present, present or referable. The reference standard used to develop and validate the DLS was the presence of either referable DR or referable DME as per the final manual human grade which was based on all captured images per patient eye.

### Automated data curation

The pool of captured images comprised of anterior segment, grayscale and ungradable samples. A small number of images also had missing laterality data (11%). An automated data curation pipeline was implemented to select the best quality two-field macula- and optic disc-centred fields from the initial pool of captured images per eye (Fig. 1B). The process addressed the identified challenges of this type of community screening via the development and testing of four independent deep learning-based models for fundal, laterality and field detection (macula and optic disc), as well as gradeability scoring (Supplementary Fig. S2)^23^. A subset of retinal photographs from the initial pool of captures images were manually graded for these parameters by a trained ophthalmologist and used to develop the deep learning curation models (for details about implementation, training and independent validation of the curation models, see Supplementary Methods and Supplementary Figs. S3, S4 and Table S3). After the removal of grayscale/non-fundus images and detection of laterality, macula and optic disc fields were identified and the image with highest gradeability score from each field per eye was selected. Eyes with an eligible pair of two-field images were selected for referable DR DLS development.

### Model development

A DLS was developed to detect referable DR/DME in a patient eye from a pair of macula and optic disc-centred handheld non-mydriatic retinal photographs (Fig. 1C). Each field was fed into an independent CNN with trainable parameters. Feature maps generated by each architecture were concatenated after a global average pooling layer (1 × 1024) and forwarded to the final fully-connected layer. All models took 766 × 578 pixel colour fundus photographs as inputs and provided an output probability for the presence of referable DR and/or DME. Higher resolution inputs up to 1149 × 867 pixel size were also investigated, but no significant improvements in DLS performance were observed.

The model encoding sections use ResNet34 architectures^24^ and were pre-trained on the ImageNet database and trained on the SM1 and SM2 datasets with five times cross-validation with fold stratification by database and DR score (SM1, SM2 and DR scores equally distributed throughout all folds), and eyes from the same patient were never part of the training set and the test set. Images are pre-processed by subtracting the local average colour and normalizing images at the channel level to ImageNet mean and standard deviation. The models were trained for 10 epochs, with a batch size of 16, and 10^–4^ initial rate with a decay factor of 0.95. Data augmentation was used in the training phase (random Gaussian blur with 5% probability, random flip with 50% probability, ± random 5% scaling, ± 10° random rotation, up to 5% random translation, and random up ± 5% shearing).

### Statistical analysis

We evaluated the ability of the DLS to predict referable DR/DME from handheld non-mydriatic retinal photographs using the area-under-the receiver operating characteristic curve (AUROC) with 1000 times bootstrapped confidence intervals (see Supplementary Methods). Additionally, we examined model sensitivity and specificity at three operating points (OP): Youden’s index (threshold defined by Eq. (1))^25^, high sensitivity (threshold defined by Eq. (2) with $\alpha =0.3$) and high specificity (threshold defined by Eq. (2) with $\alpha =0.7$).

1 $${t}_{Y}=argma{x}_{t}J\left(t\right), \;\; where \;\; J\left(t\right)=[sensitivity\left(t\right)+specificity \left(t\right)-1]$$

2 $$t=argma{x}_{t} f\left(\alpha \right), \;\; where \;\; f\left(\alpha \right)= [\alpha *Specificity\left(t\right)+\left(1-\alpha \right)*Sensitivity(t)]$$

Inter-grader agreement between primary and secondary graders, and between final grades (after arbitration) and primary and secondary graders, respectively, were calculated with exact Clopper-Pearson CIs with 95% confidence levels.

DLS performance was compared to the prognostication obtained by using individual-level risk factors. Univariable and multivariate logistic regression models were trained using available risk factors to identify the presence of referable DR/DME in either eye. Univariate models were trained using glycated haemoglobin levels (HbA_1c_), duration of diabetes, systolic and diastolic blood pressure, and body mass index (BMI). Multivariate models included systolic and diastolic blood pressure alone and all aforementioned risk factors.

## Results

From a pool of 81,320 retinal fundus images, a total of 32,494 images from 16,247 eyes (9778 individuals) were eligible for the study (Supplementary Fig. S2), comprised of a pair of macula-centred and optic disc-centred images for each person eye. Participant demographics and distribution of the DR grades for both SM1 and SM2 cohorts are shown in Table 1. In SM1, the average age of the participants was 54.40 (10.72) years, with 49.02% males, 4.70% DR referable eyes, and 3.20% DME referable eyes. In SM2, the average age of the participants was 55.38 (9.28) years, with 66.95% males, 88.75% DR referable eyes, and 60.55% DME referable eyes.

**Table 1:** Summary of data characteristics. ^1^Random blood sugar > 160 mg/dl and HbA1c > 6.5% at screening.

	SMART-India 1	SMART-India 2
**Dataset characteristics**		
Number of participants	7288	2490
Number of eyes	12,708	3539
Number of images per eye	2-field (macula, optic disc)	2-field (macula, optic disc)
Ethnicity	Indian	Indian
**Patient demographics**		
Age, years	54.40 (10.72)	55.38 (9.28)
Gender		
Female	3714 (50.96%)	823 (33.05%)
Male	3573 (49.02%)	1667 (66.95%)
Other	1 (0.01%)	0 (0.00%)
Smokers	391 (5.36%)	91 (3.65%)
**Systemic risk factors**		
Body-mass index	26.06 (4.55)	25.06 (3.51)
Median diabetes duration, years	5 (0–60)	12 (0–46)
Blood pressure, mmHg		
Systolic	137.10 (21.29)	141.31 (19.54)
Diastolic	85.06 (11.98)	84.44 (10.44)
Diabetes status		
Diabetic	4121 (56.55%)	2490 (100.0%)
Unknown^1^	3167 (43.45%)	0 (0.00%)
HbA_1c_, %	7.86 (2.23)	8.47 (1.94)
**Eye characteristics**		
Non-referable DR	12,111 (95.30%)	398 (11.25%)
No DR	11,563 (90.99%)	214 (6.05%)
Mild non-proliferative DR	548 (4.31%)	184 (5.20%)
Referable DR	597 (4.70%)	3141 (88.75%)
Moderate non-proliferative DR	480 (3.78%)	1401 (39.59%)
Severe non-proliferative DR	65 (0.51%)	661 (18.68%)
Proliferative DR	52 (0.41%)	1079 (30.49%)
Diabetic macular oedema	407 (3.20%)	2143 (60.55%)

**Figure 2:**
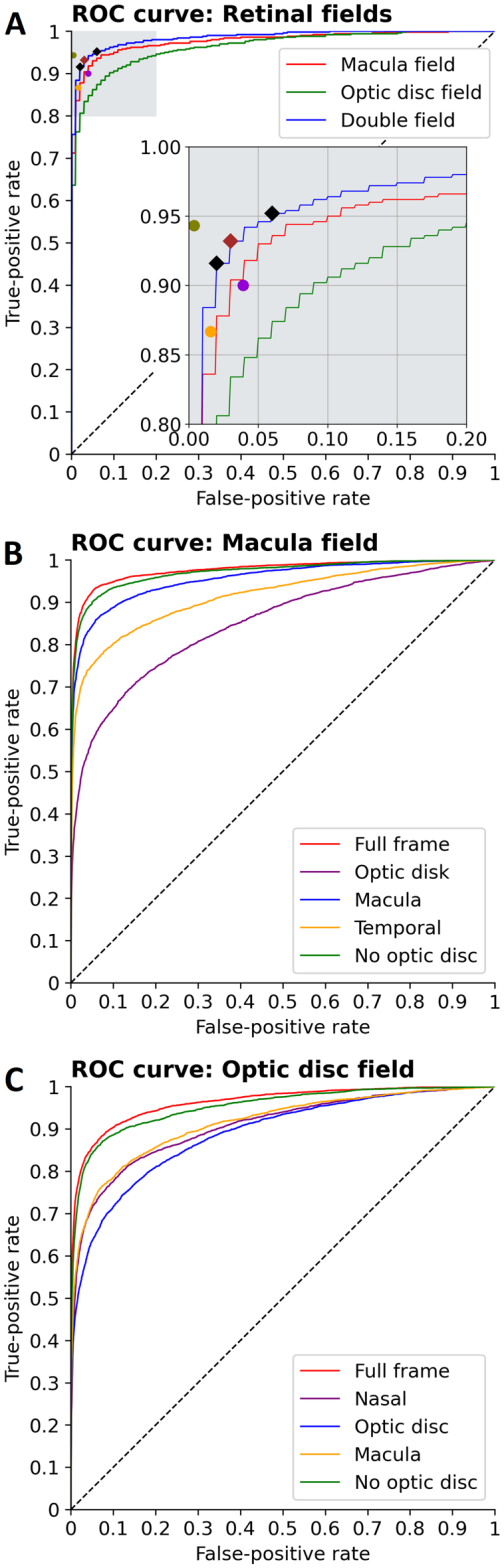
ROC curves. (**A**) ROC curves for different field inputs. Close up shows operating points (red: Youden’s index OP, black diamonds: high sensitivity and high specificity OPs) and intergrader values (purple: primary vs. secondary, orange: primary, green: secondary) depicted. (**B**) ROC curves illustrating algorithm performance based on different input regions from the macula field. (**C**) ROC curves illustrating algorithm performance based on different input regions from the optic disc field.

The AUROC of the DLS for referable DR/DME was 0.985 (1000 times bootstrapped 95% CI 0.98–0.99) (Fig. 2A). When the prediction was obtained with a single field, the AUROCs was 0.977 (0.98–0.98) for the macula field (Fig. 2B) and 0.963 (0.95–0.98) for the optic-disc field (Fig. 2C). When evaluated independently in SM1 and SM2, the DLS achieved 95.47 (91.75–97.98) and 95.27 (92.74–97.14) AUROC, respectively.

**Figure 3:**
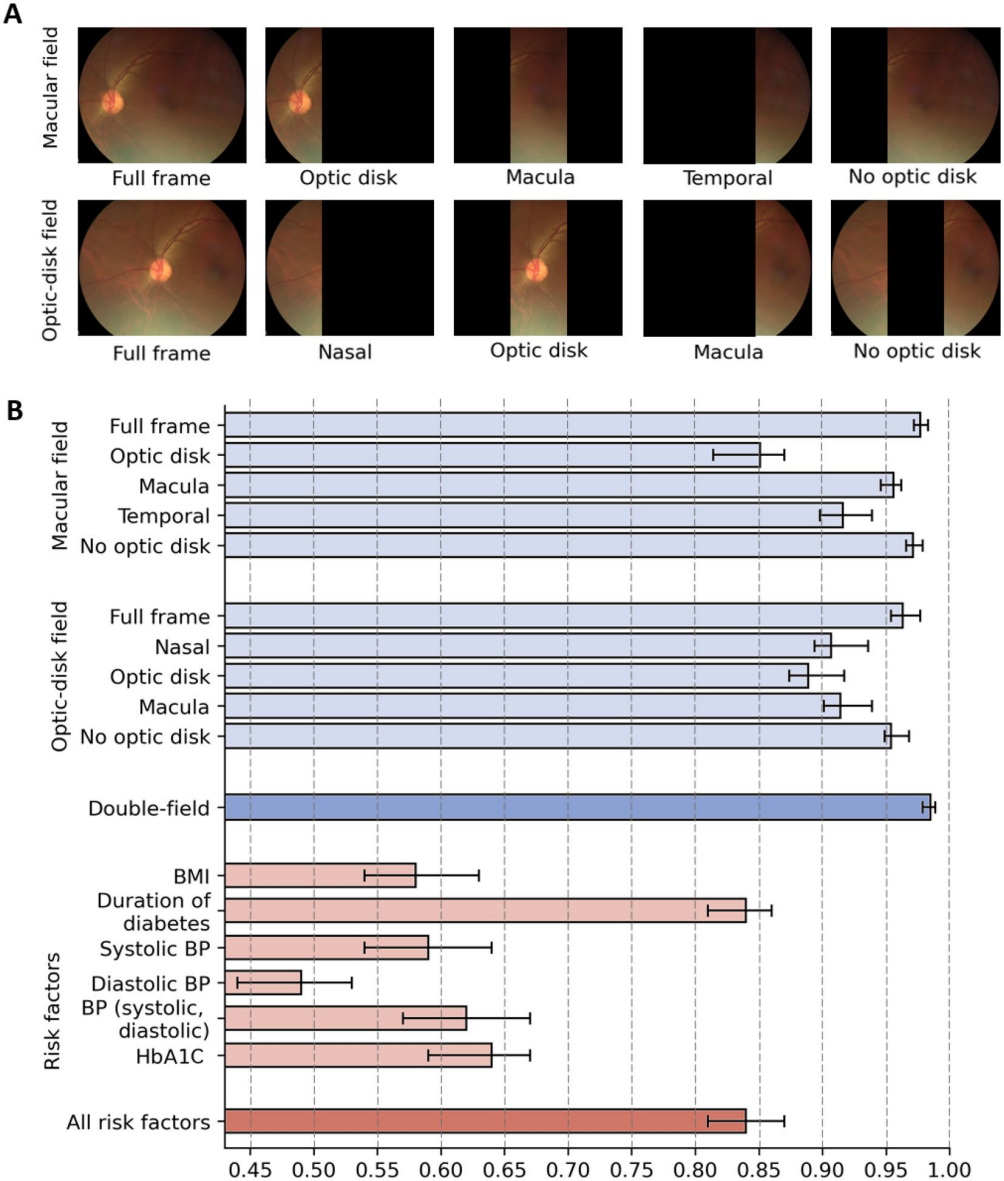
Assessment of different fields and parts of the images. (**A**) Illustration of image regions evaluated in the input ablation study. (**B**) DLS performance achieved by each image region from the macula field and the optic disc field (Supplementary Table S1) and by univariate/multivariate analysis of person level risk factors (Supplementary Table S2). Error bars indicate 1000 times bootstrap 95% CIs.

To assess region importance in referable DR/DME prediction, we evaluated DLS performance with input ablation (Fig. 3, Supplementary Table S1). Both retinal fields where vertically split in three regions. For the macula field, AUROC for the macula region was 0.96 (1000 times bootstrapped 95% CI 0.95–0.96), decreasing to 0.92 (0.90–0.94) for the temporal region, and 0.85 (0.81–0.87) for the optic disc region. For the optic-disc field, AUROC reached 0.91 (0.90–0.94) for the macula region, 0.91 (0.89–0.94) for the nasal region, and 0.89 (0.87–0.92) for the optic disc region. When only the region corresponding to optic disc was occluded, AUROC reached 0.97 (0.97–0.98) for the macula field, and 0.95 (0.95–0.97) for the optic disc field.

Sensitivity and specificity of three different OPs of the DLS were examined and compared to inter-grader performance (Table 2). Youden’s OP reached 93.86% (1000 times bootstrapped 95% CI 91.34–96.08) sensitivity and 96.00% (94.68–98.09) specificity. The high sensitivity OP corresponded to 95.53% (93.64–97.41) sensitivity and 92.79% (88.96–97.40) specificity, and the high specificity OP reached 90.88% (88.80–95.23) sensitivity and 98.00% (96.29–98.88) specificity. Primary grader agreement with final grades (after arbitration when disagreement between primary and secondary grades) reached 86.67% (85.52–87.75) sensitivity and 98.43% (98.18–98.64) specificity, whilst secondary graders agreement with final grades showed 94.32% (93.52–95.05) sensitivity and 99.63% (99.50–99.73) specificity. Primary graders vs. secondary graders reached 90.01% (88.95–91.01) sensitivity and 96.09% (95.72–96.42) specificity.

**Table 2:** Intergrader and algorithm performance at different operating points.

	Sensitivity	Specificity
Primary vs. secondary	90.01 (88.95–91.01)	96.09 (95.72–96.42)
Primary grades	86.67 (85.52–87.75)	98.43 (98.18–98.64)
Secondary grades	94.32 (93.52–95.05)	99.63 (99.50–99.73)
DLS (Youden’s J)	93.86 (91.34–96.08)	96.00 (94.68–98.09)
DLS (high sensitivity)	95.53 (93.64–97.41)	92.79 (88.96–97.40)
DLS (high specificity)	90.88 (88.80–95.23)	98.00 (96.29–98.88)

For intergrader agreement on referable DR detection, gradable person eyes for both primary and secondary graders are considered. In all cases, with the exception of Primary vs. Secondary intergrader assessment, Primary and secondary grade agreement with the reference standard (after arbitration) is reported.

Univariate analysis of person level risk factors (Fig. 3, Supplementary Table S2 in the Supplement) showed the duration of diabetes had the most significant predictive association, with an AUROC of 0.84 (1000 times bootstrapped 95% CI 0.81–0.86), followed by the glycated haemoglobin (HbA1c) levels, with an AUROC of 0.64 (0.59–0.67). Multivariate analyses of duration of diabetes, glycated haemoglobin levels, systolic and diastolic blood pressure (BP), and BMI reached 0.84 (0.81–0.87).

**Figure 4:**
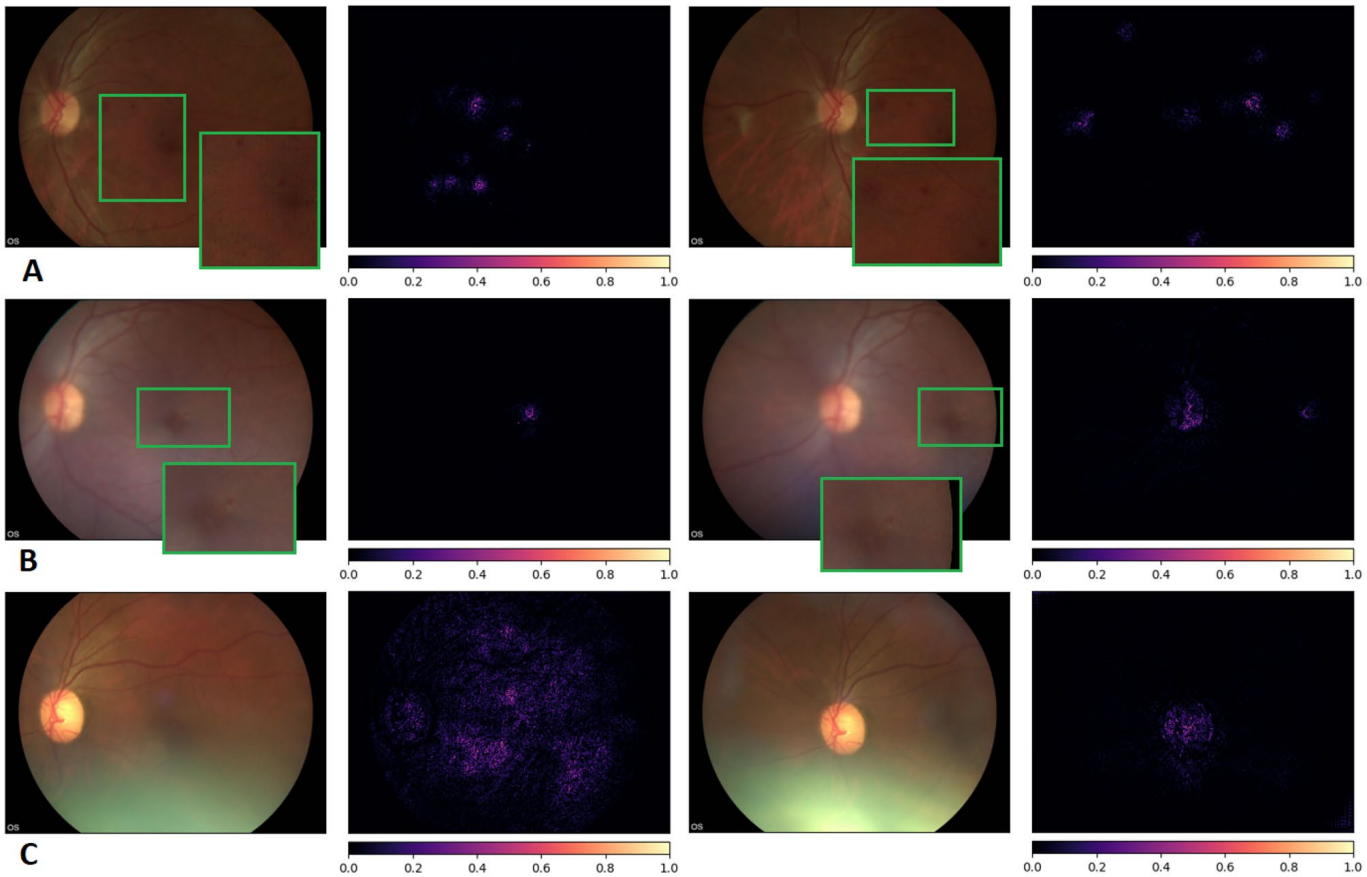
Integrated gradients pixel attributions. Pairs of macula and optic disc centred images of three different patient eyes and the obtained saliency maps (**A**) Severe NPDR, referable DME, DLS score 0.98. (**B**) Moderate NPDR, No DME, DLS score 0.58, (**C**) No DR, No DME, 0.04. The saliency maps highlight relevant lesions for the diagnosis (microaneurysms). In the absence of referable DR/DME, only the optic disc or general regions of the retina are highlighted.

Integrated gradients were used to gain insight into the retinal features learned by the DLS^26^. The saliency maps highlight the most influential pixels in the DLS decision (Fig. 4). When signs of referable DR/DME are present in the image (Fig. 4A,B) the parts of the image where the specific lesions are located (e.g. microaneurysms) are prominently highlighted. The DLS consistently highlights lesions even when they are hardly visible to the naked eye. In the absence of referable DR/DME, only the optic disc or general regions of the retina are highlighted.

## Discussion

The use of handheld non-mydriatic retinal images for screening poses unique challenges for automated DR detection systems due to variable image quality. The majority of prior automated referable DR detection systems have been developed using guidelines and acquisition conditions reflective of high-income countries^15,17,27^. The application of these systems to handheld, non-mydriatic retinal images results in significantly reduced model performance^19^. Therefore, there is a need to develop and evaluate automated grading systems using screening and acquisition conditions matching those found in LMIC. Evidence of the efficacy and applicability of automated DR detection systems in resource limited environments could greatly widen the availability of DR screening which, in turn, could help reduce preventable sight loss. In this study, we developed and validated a DLS that achieved a clinically acceptable level of performance in detecting referable DR and DME from handheld, non-mydriatic retinal images acquired in community settings by field workers in India, a LMIC.

Prior to the advent of deep learning-based techniques, feature-based approaches had been explored to assist on the screening of DR from different retinal image modalities^28,29^. Detection frameworks based on geometric features, vessel analysis and retinal hemodynamics had been widely studied^30,31^. However, in recent years, deep learning success at classification tasks has paved the way for new achievements in the automated diagnosis of referable DR. Several studies have recently explored the detection of referable DR using deep neural networks^15,17,27^. These studies used mydriatic retinal photographs acquired from in-clinic screening programs by imaging professionals using table-top retinal cameras. Gulshan et al.^27^ evaluated a deep learning system for referable DR reporting a sensitivity of 90.3% and a specificity of 98.1% specificity for the EyePACS-1 public dataset. Similarly, Ting et al.^17^ algorithm reported a sensitivity of 90.5% and a specificity of 91.6% on a proprietary validation dataset. More recently, a prospective study by Gulshan et al.^14^ evaluated automated DR detection reaching best performance of 92.1% sensitivity and 95.2% specificity at some sites. In their study, the authors highlighted the impact of acquisition settings (in-clinic and community-based) in algorithm performance. Bellemo et al.^15^ reported a sensitivity 99.42% for detecting vision-threatening DR and 97.19% for referable maculopathy in a study based in a LMIC (Zambia).

A few studies have also explored a more accurate classification of the DR stages. Wang et al.^32^ examined the performance of different architectures at DR staging when trained in a 166 image subset of the Kaggle dataset^33^, with InceptionNet V3^34^ reaching the best results at 63.23% accuracy. Khan et al.^35^ proposed a custom convolutional model and reported an accuracy of 98.15% dataset on the Messidor dataset^36^. Harangi et al.^37^ used the Kaggle dataset^33^ to train a framework that combined AlexNet^38^ and hand-crafted features. The authors reported an accuracy of 90.07% when tested on the IDRiD dataset^39^. Li et al.^40^ achieved a joint accuracy of 65.1% the IDRiD dataset^39^ by training a ResNet50 architecture^24^. Qureshi et al.^41^ proposed a framework trained on EyePACS-1 dataset based on patch extraction and classification and reported 92.20% sensitivity and 95.10% specificity. Alyoubi et al.^42^ with a custom convolutional model, achieved an accuracy of 88.6% and 84.1% on the DDR^43^ and the APTOS Kaggle 2019 dataset^44^, respectively.

Few studies have explored DLS performance using handheld retinal images or community-based settings. Notable exceptions are Rajalakshmi et al.^45^ who reported a sensitivity of 95.8% and a specificity of 80.2% at detecting any DR using 2408 smartphone-based mydriatic fundus images acquired by hospital trained staff in clinic environment. A pilot study by Natarajan et al.^46^ on 223 patients with diabetes (PwD) reported 100.0% sensitivity and 88.4% specificity for referable DR detection using a smartphone-based automated system. Sosale et al., in a prospective study including 922 individuals, developed a smartphone-based system using a combination of non-mydriatic and mydriatic images acquired in clinical settings by a trained camera technician. Their referable DR system using pairs of macula and disc centred images reported 93.0% sensitivity and 92.5% specificity.

There are differences between these studies and ours. In our study, we developed a fully automated DLS to detect referable DR/DME in a setting that mirrors real-life implementations in LMIC. We evaluated the DLS system on handheld non-mydriatic retinal photographs acquired by field workers and demonstrated competitive or better performance to prior studies despite the unfavourable acquisition conditions. Clinically acceptable performance was achieved by the DLS either using a two-field (macula and optic-disc centred) or single-field inputs independently, most notably with macula only images. As retinal screening is not available in many countries worldwide, we also evaluated the predictive performance of different risk factors available by training univariate and multivariate logistic regression models and assessing their comparative predictive performance. Among the different risk factors we studied, duration of diabetes had the highest predictive significance. Adding other risk factors had no additional contribution in the multivariate model. The image based DLS outperformed all risk factor-based models in detecting referable DR/DME, highlighting the need to establish retinal screening programmes globally. Incorporating this fully automated DLS in low-cost cameras is likely to reduce the healthcare burden of DR screening worldwide.

As image quality is suboptimal and only some areas of the 2-field images may be missing, we also carried out a comprehensive set of image region ablation studies to better understand the contribution of different images areas to the prediction. The findings showed that the optic-disc regions, both within the macula field and the optic-disc field, had the lowest significance. This is evident from the fact that DLS performance was slightly reduced when only the optic disc area was occluded, whilst using the optic disc region alone yielded the lowest performance. On the other hand, the inclusion of the macula area achieved the highest performance compared to each of the other independently evaluated regions. These findings have significance, as image occlusion is likely to occur in a non-trivial proportion of images captured using non-mydriatic handheld cameras. Hence, we could demonstrate likely impacts on performance. Our results show that people with DME are more prevalent than severe DR, which is why model performance is significantly impacted with the occlusion of the macula field. Optic disc neovascularisation is a sight threatening complication that requires treatment. Therefore, despite the challenges of capturing 2-field images through non-mydriatic pupils, it is crucial for field workers to be trained to obtain both the macula and optic disc field images. Obtaining the optic disc field alone without macular field is likely to miss significant numbers of referable DR/DME.

Manual grading was performed by independent primary and secondary graders. In case of discrepancies, the grading was arbitrated by a senior consultant who had access to the primary and secondary grades. We evaluated intergrader performance and compared it to the deep learning system performance at different operating points. A significant difference in sensitivity was found when evaluating primary grader and secondary grader agreement to the final grades (after arbitration). The lower sensitivity by primary graders showed a higher restrictive standard at detecting referable cases. Three different operating points of the DLS were evaluated. The high specificity point performance aligned closely with that of human graders. Whilst the balanced operating point (maximising Youden’s index) reached a sensitivity comparable to the best intergrader values with a preserved level of specificity. Overall, the findings highlight comparable performance between human graders and the DLS.

To the best of our knowledge, this is the first prospective multi-centre study mirroring a real-life implementation of DR screening in a LMIC and includes a considerably large dataset of handheld retinal images taken by field workers in a community setting. Our results demonstrate that these photographs can be used to develop deep learning-based systems capable of detecting referable DR. Our findings can contribute to the development of novel screening guidelines supported by deep learning systems and guide policy makers in establishing new scalable, cost-effectively approaches to detect vision threatening retinopathy in countries with low resources, where most of the PwD reside.

Our study has some limitations. First, the study is based on a mono-ethnic population. Hence, different fundal appearance and DR/DME expression in any other ethnicities may affect algorithm generalization and, therefore, influence performance when applied to different populations. Second, the pool of retinal photographs acquired by the field workers required curation to discard incorrectly acquired images and select gradable 2-field images suitable for referable DR DLS development. The deployment of DR screening programs in LMIC and the employment of non-technical field workers makes this curation pipeline a necessary step prior to DR/DME screening. Our demonstrates that this limitation can be addressed with deep learning techniques and be successfully automated. Third, manual graders had access to all the retinal images acquired for each patient eye and provided their decision on referable DR/DME whereas the DLS, limited to a finite number of input photographs, provided predictions on the basis of the pair of resulting images from this curation process, which could possibly include outliers (e.g., misclassified field images). However, the impact of outliers resulting from the curation process is considered small given that the deep learning algorithms involved in the curation process showed excellent performance for each of the curation tasks (see Supplementary Methods).

In conclusion, our study highlights the efficacy of automated deep learning-based detection of referable DR and DME using handheld non-mydriatic retinal images in community settings. Our findings have particular relevance for policy makers in LMIC aiming to implement cost-effective, scalable and sustainable DR screening programmes.

## Supplementary Information

Supplementary Information.

## Data Availability

The datasets used and/or analysed during the current study are available from the corresponding author on reasonable request.
