# Supplementary material for: Using deep learning to detect diabetic retinopathy on handheld non-mydriatic retinal images acquired by field workers in community settings
Source: Sci Rep. 2023 Jan 25;13:1392. doi: 10.1038/s41598-023-28347-z (PMC9876892; doi:10.1038/s41598-023-28347-z)
Supplement: Supplementary file 1 — Supplementary Information. [file 41598_2023_28347_MOESM1_ESM.docx]

**Supplementary Materials**

**Using deep learning to detect diabetic retinopathy on handheld non-mydriatic retinal images acquired by field workers in community settings**

Joan M. Nunez do Rio PhD, Paul Nderitu FRCOphth, Rajiv Raman MS, Ramachandran Rajalakshmi FRCS PhD, Ramasamy Kim DO DNB, Padmaja K Rani MS FRCS, Sobha Sivaprasad FRCOphth, Christos Bergeles PhD for the SMART India Study Group. Using deep learning to detect diabetic retinopathy on handheld non-mydriatic retinal images acquired by field workers in community settings.

**Table of Contents**

[Supplementary Methods 2](#_Toc122389189)

[Dataset curation 2](#_Toc122389190)

[Dataset curation models 2](#_Toc122389191)

[Statistical analysis 2](#_Toc122389192)

[Supplementary Figures 3](#_Toc122389193)

[Figure S1 3](#_Toc122389194)

[Figure S2 4](#_Toc122389195)

[Figure S3 5](#_Toc122389196)

[Figure S4 6](#_Toc122389197)

[Figure S5 7](#_Toc122389198)

[Supplementary Tables 8](#_Toc122389199)

[Table S1 8](#_Toc122389200)

[Table S2 8](#_Toc122389201)

[Table S3 8](#_Toc122389202)

[Table S4 9](#_Toc122389203)

[Table S5 9](#_Toc122389204)

[Supplementary References 10](#_Toc122389205)

# Supplementary Methods

## Dataset curation

Hand-held non-mydriatic retinal photographs collected by field workers in 20 sites around India (see Supplementary Fig. 1S) were independently graded at the local clinical centre (primary grading) and transferred to four central reading centres for secondary grading. A centralised cloud-based database stored anonymised collected data, retinal photographs and manual grades (primary, secondary and arbitration/final). Collected data, indexed by patient after collection and during the grading process, was subsequently curated via an automated process to generate two-field datasets of patient eyes, i.e., a pair of macula-centred and optic-disc-centred images for each patient eye. Four deep learning models were developed to perform the data curation: colour fundus image detection, eye laterality detection, field detection (macula & optic disc) and gradeability scoring.^1^ See Supplementary Fig. S2 for a quantitative illustration of the process.

Collected retinal photographs (60,633 for SMART-India1, SM1, and 20,737 for SMART-India2, SM2) went through a sequential process to curate the final datasets:

1. Colour retinal fundus images were selected as identified by the fundus/non-fundus detection model with anterior segment and grayscale images discarded. See receiver operating characteristic (ROC) curve and precision-recall curve (PRC) in Supplementary Fig. S3. Images with a missing patient index or DR/DMO grade were also excluded at this stage.
2. Laterality was then identified for images missing this data (11%) using the laterality model (see ROC and PRC in Supplementary Fig. S3). After this stage it was possible to identify all retinal photographs by patient eye.
3. Gradable patient eyes based on manual grades were then selected (23,386 gradable patient eyes from 12,538 patients). It should be noted that as manual labels identified gradeability at the eye level, ungradable images could exist within the pool of images as long as one or more images was gradable per eye.
4. The retinal field detection model was then used to identify macula and optic-disc field images. At this stage, a variable number of images from either field but of potentially varying quality were present.
5. The highest gradeability prediction derived using the gradeability model was used to select the ‘best’ macula and optic-disc image from a set of images per eye (see ROC and PRC for both models in Supplementary Fig. S4).
6. Patient eyes including both a macula and optic-disc-centred images were selected. Those with only either field exclusively were excluded.

## Dataset curation models

The Fundus/non-fundus, Laterality, Retinal field, and Gradeability models were trained and evaluated on a subset of SM1 images (Supplementary Table S3 for dataset information for each specific model). The identical CNN architectures comprised an EfficientNet-V1-B0^2^ followed by two Conv-BN-dropout blocks and a dense layer. All models were five times cross-validated with fold stratification by patient so that eyes from the same patient were never part of the training set and the test set. Images were resized to 224x224, rescaled (0-1) and standardised per channel and data augmentation was used in the training phase (+/- 20% brightness, random flip with 50% probability, 0 to 25% zoom, and +/- 5 degrees random rotation). The models were initialised with weights pre-trained on ImageNet database^3^ and trained for 20 epochs, with a batch size of 16, and 10^-3^ initial learning rate with exponential decay every 3 epochs with a 10^-5^ rate.

## Statistical analysis

n times bootstrapped confidence intervals were calculated by creating n resamples (with replacement) of the set of observations and computing the metric of interest on each of the resamples. With n=1000, the ranked values of the metric of interest for all the resamples were used to determine the 95% confidence intervals by selecting as their boundaries the 25^th^ value and the 975^th^ value (2.5^th^ and 97.5^th^ percentiles, respectively).

# Supplementary Figures


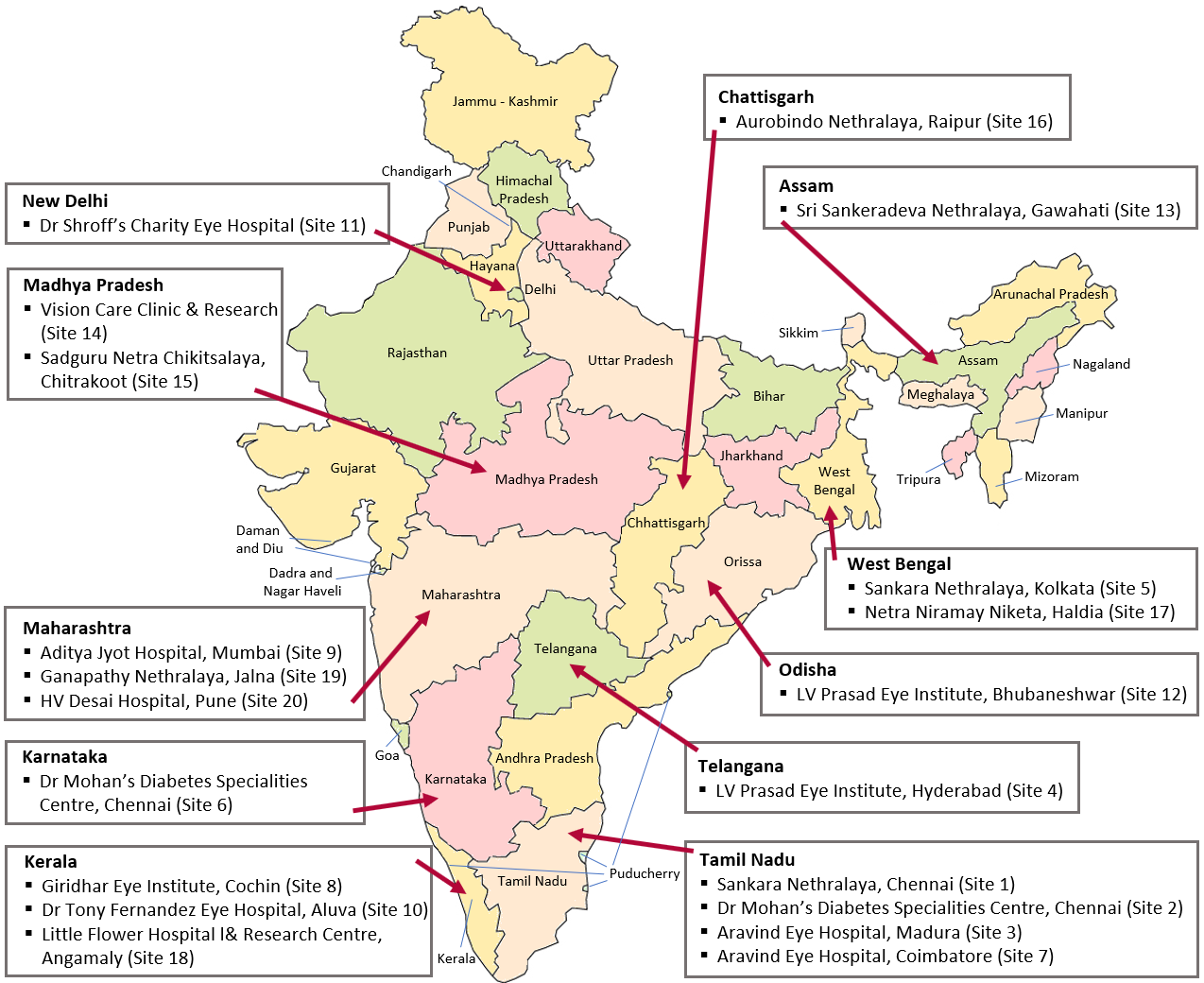


Figure S1*.* SMART-India sites (Adobe Photoshop CS6 v13.0.1 www.adobe.com). The study was conducted across 20 regions in India, each led by a local clinical centre and with a trained ophthalmologist responsible for each site.


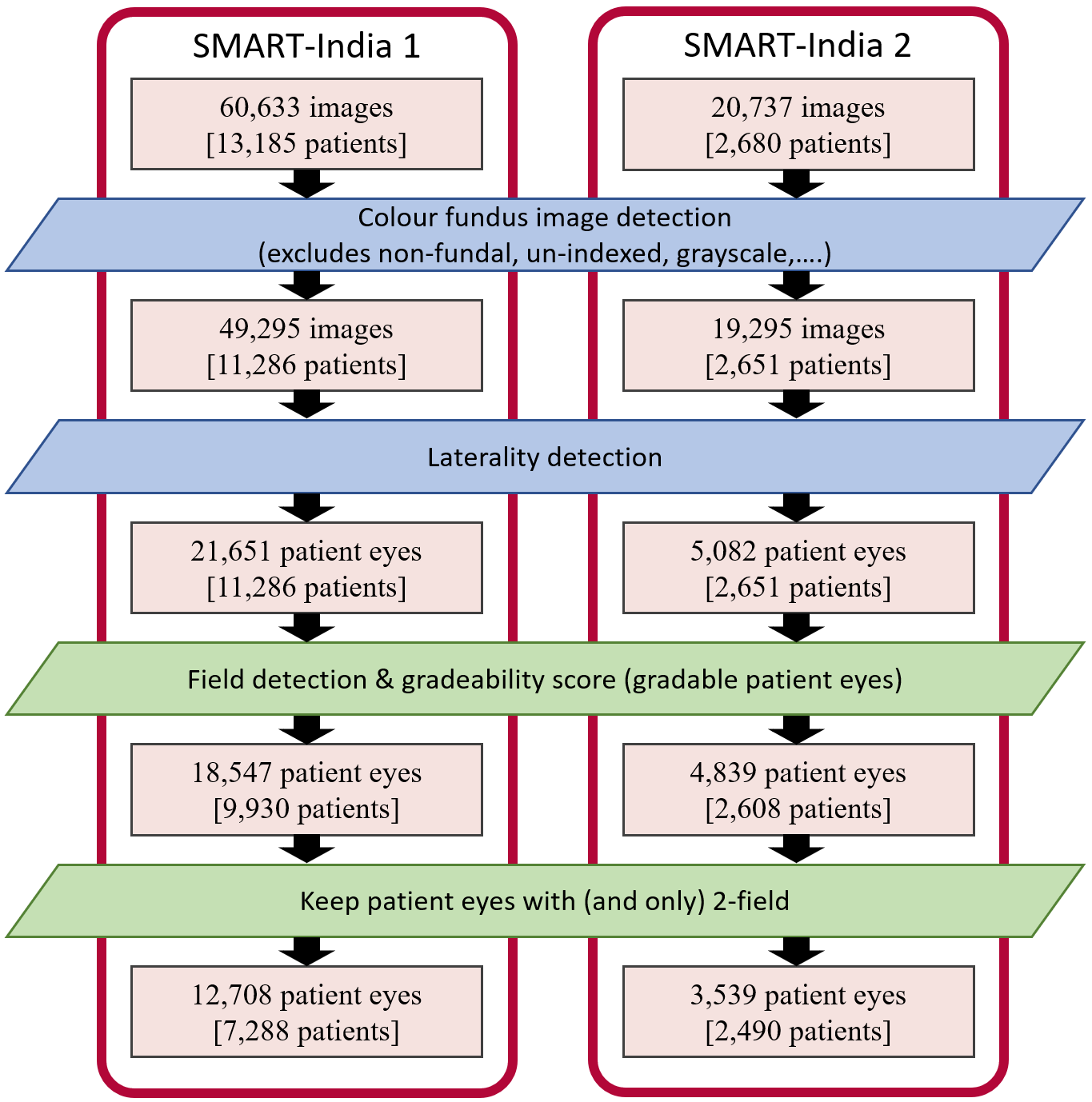


Figure S2**.** Study participants and data curation. Four deep learning models were used in the automated data curation. Gradable patient eyes were extracted after the two first steps (colour fundus image detection and laterality detection). Then, retinal fields were separated and ranked by their gradeability score. Patient eyes with a pair of macula and optic disc centred photographs were used for the development of the DLS for referable DR/DME detection.

| **Fundus image model**  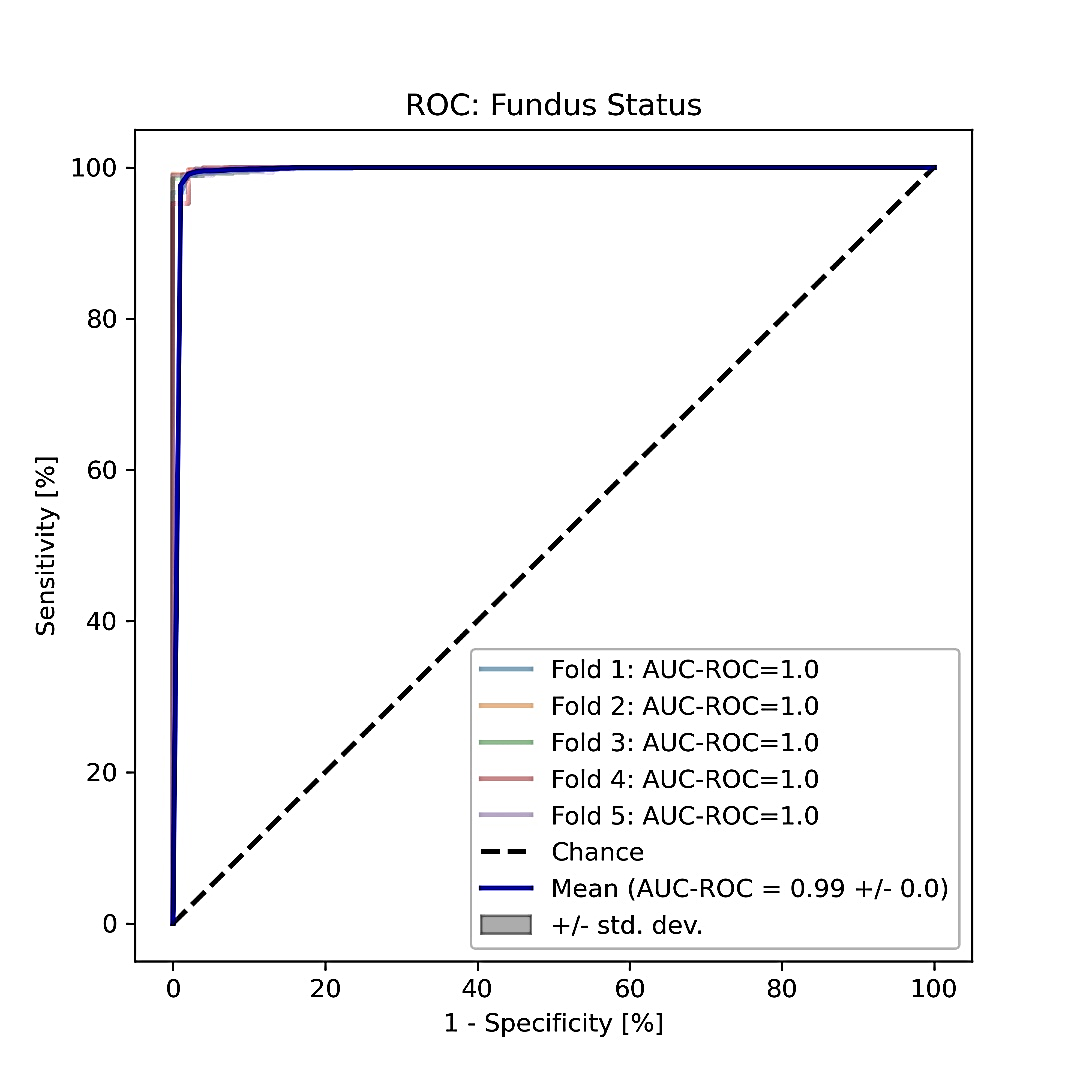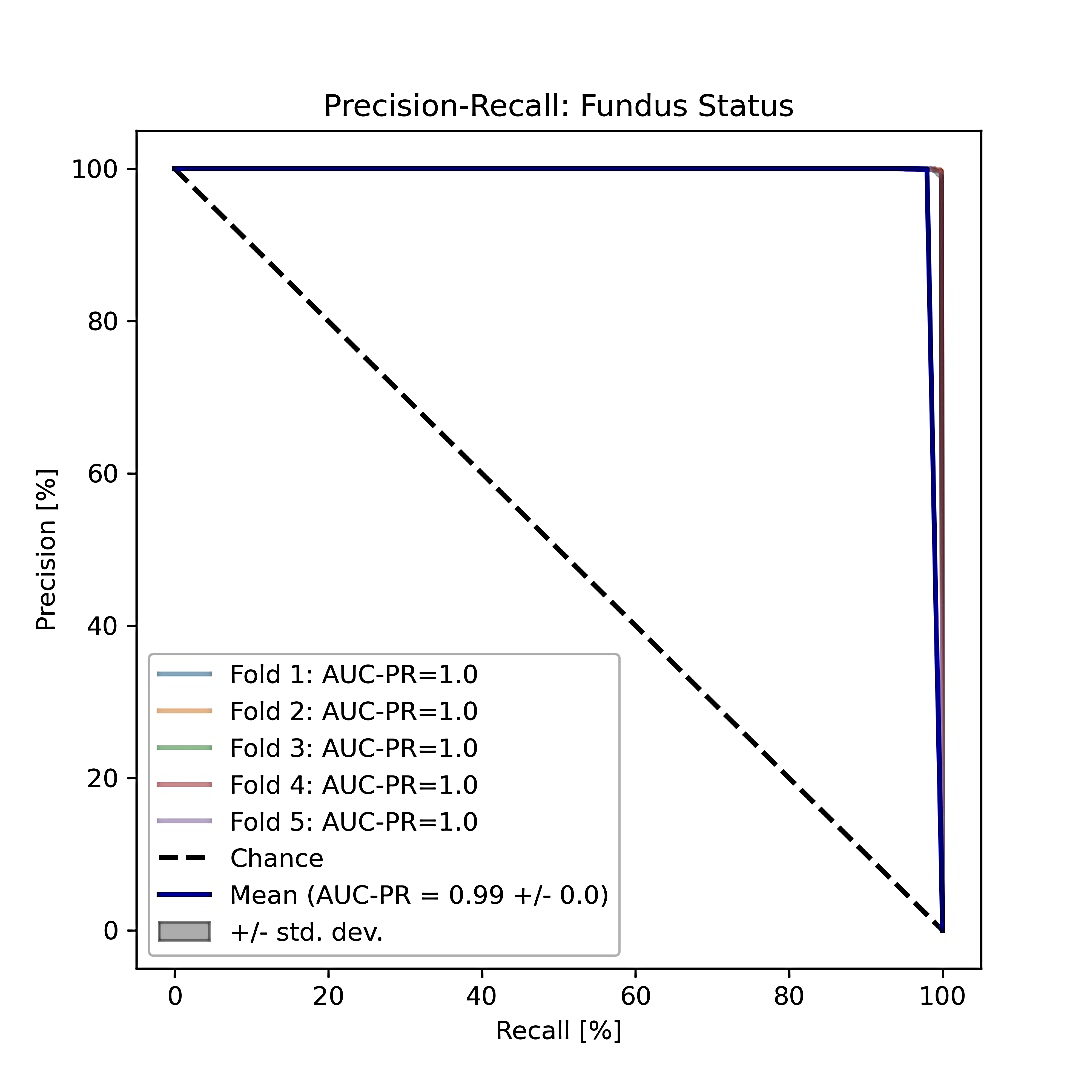 |
| --- |
| **Laterality model**  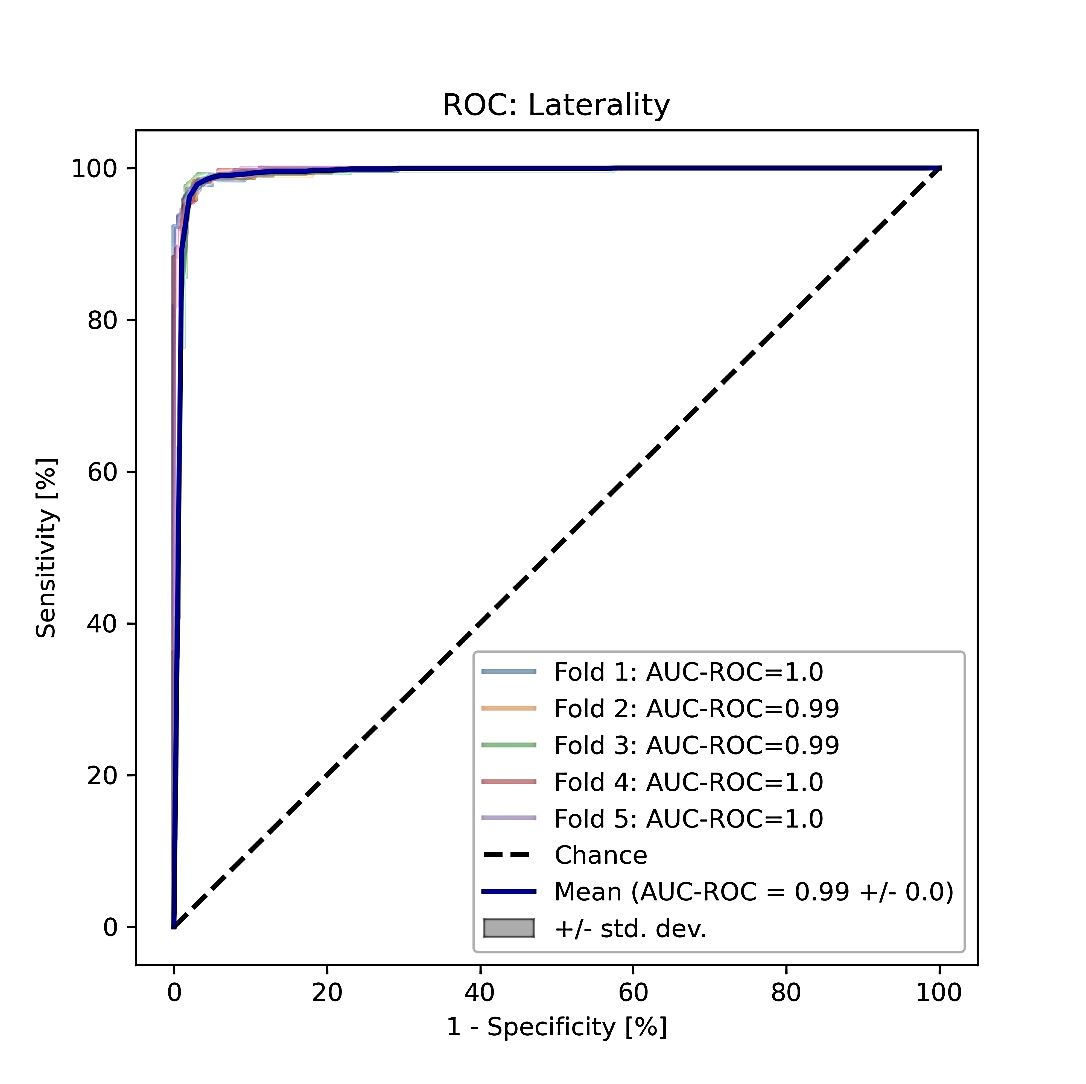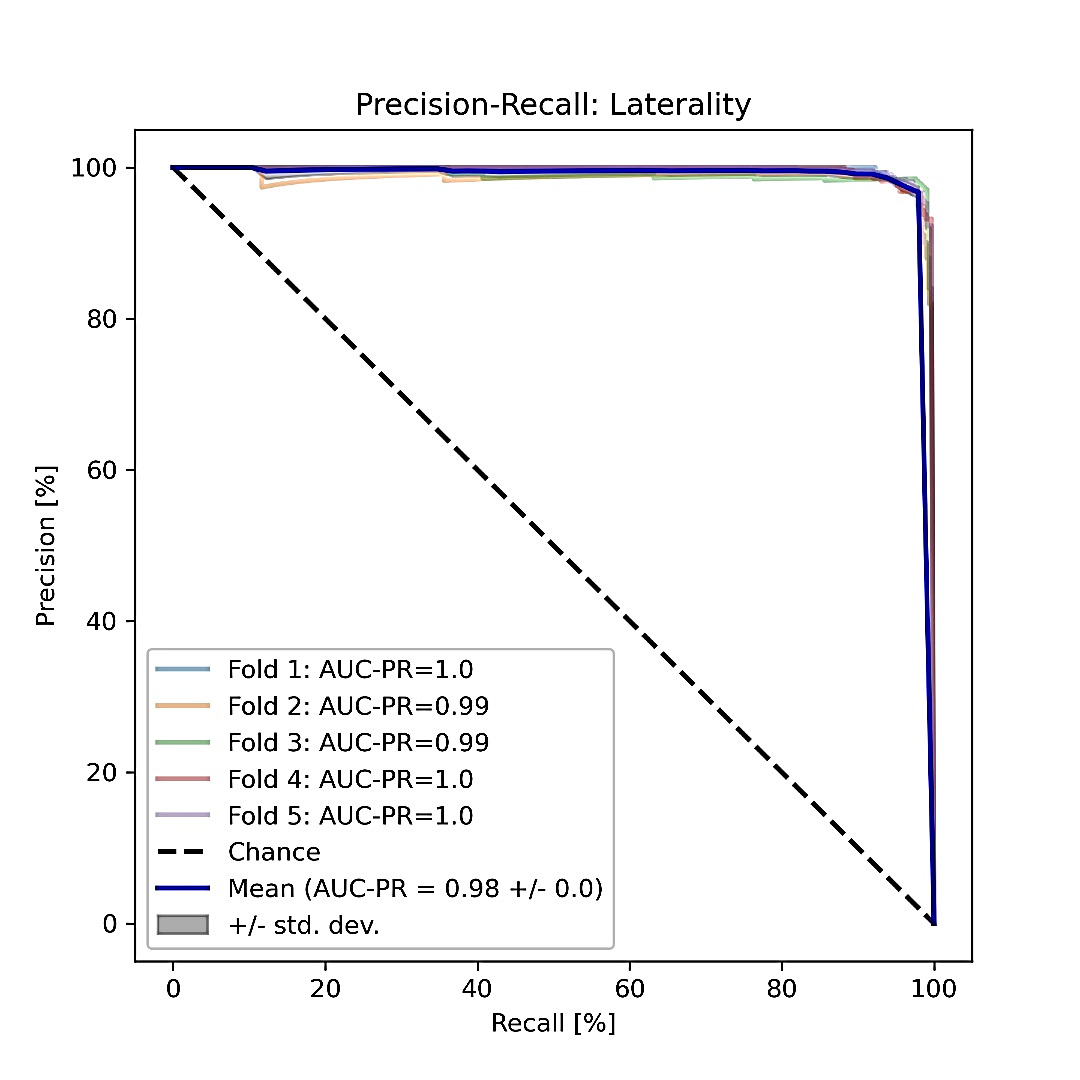 |

Figure S3**.** Data curation. Performance of curation models for fundus image detection and laterality detection.

| **Field model**  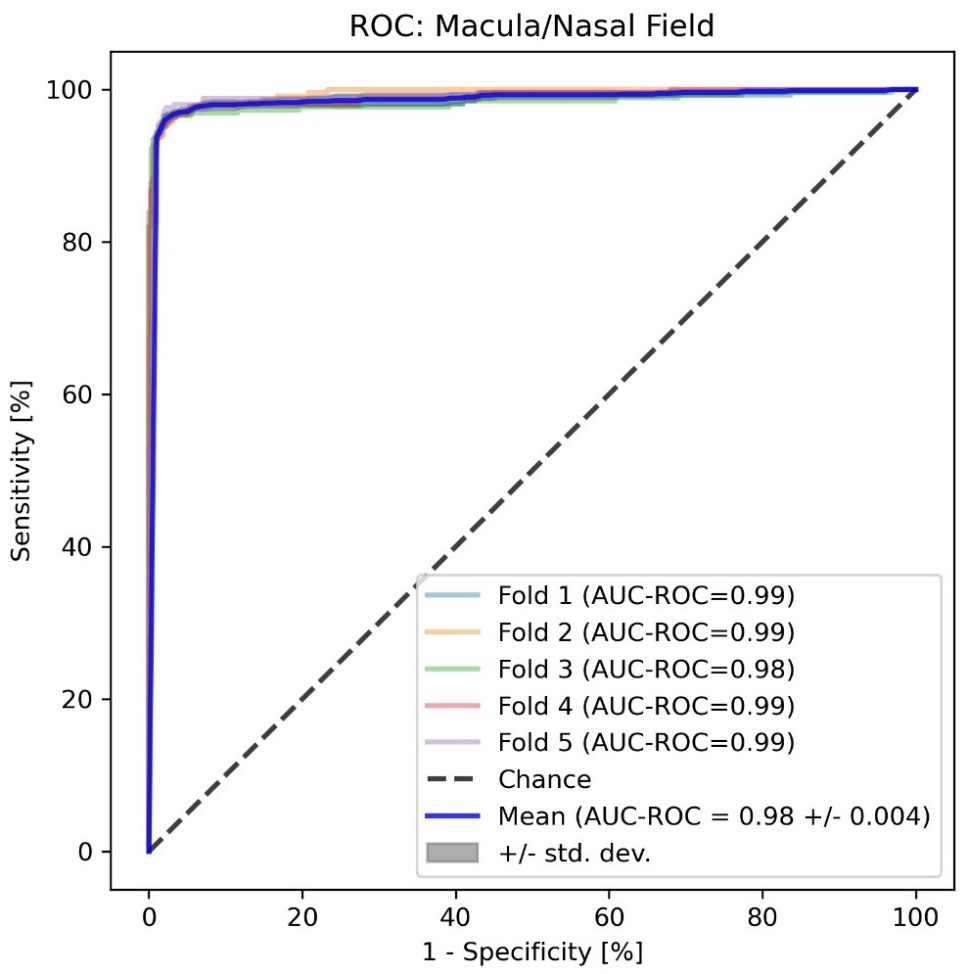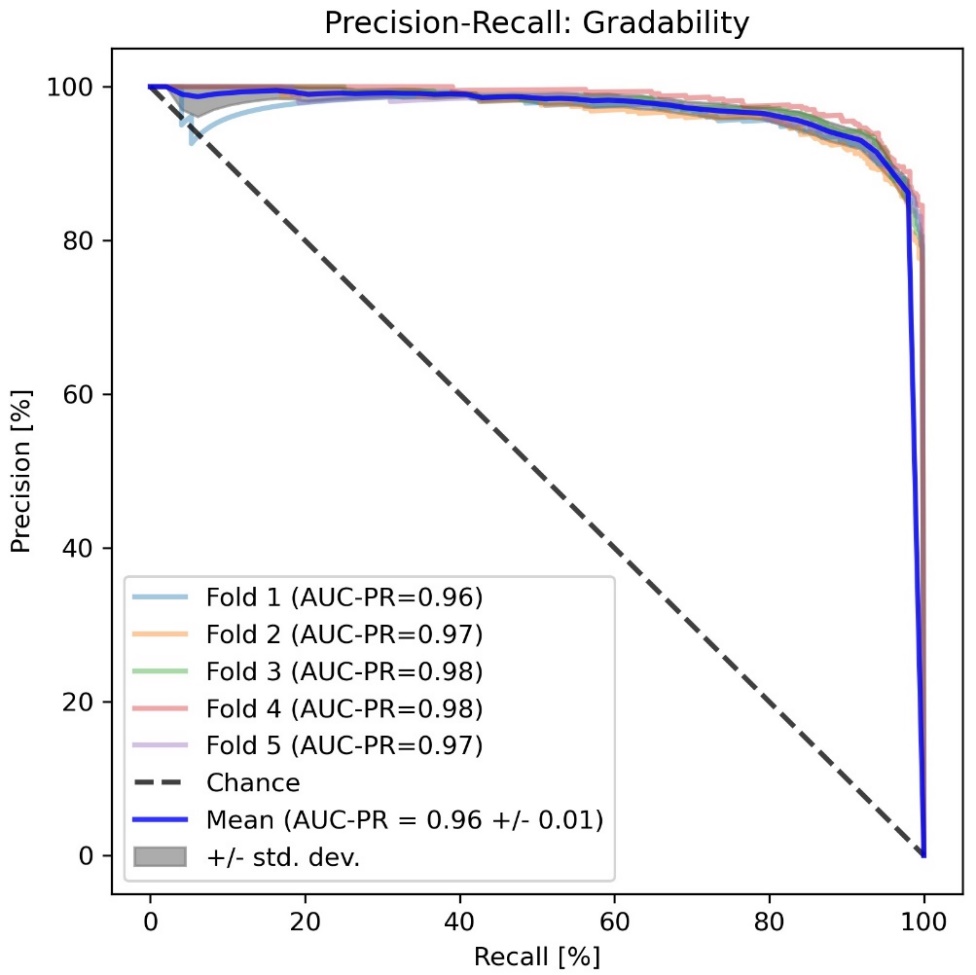 |
| --- |
| **Gradeability model**  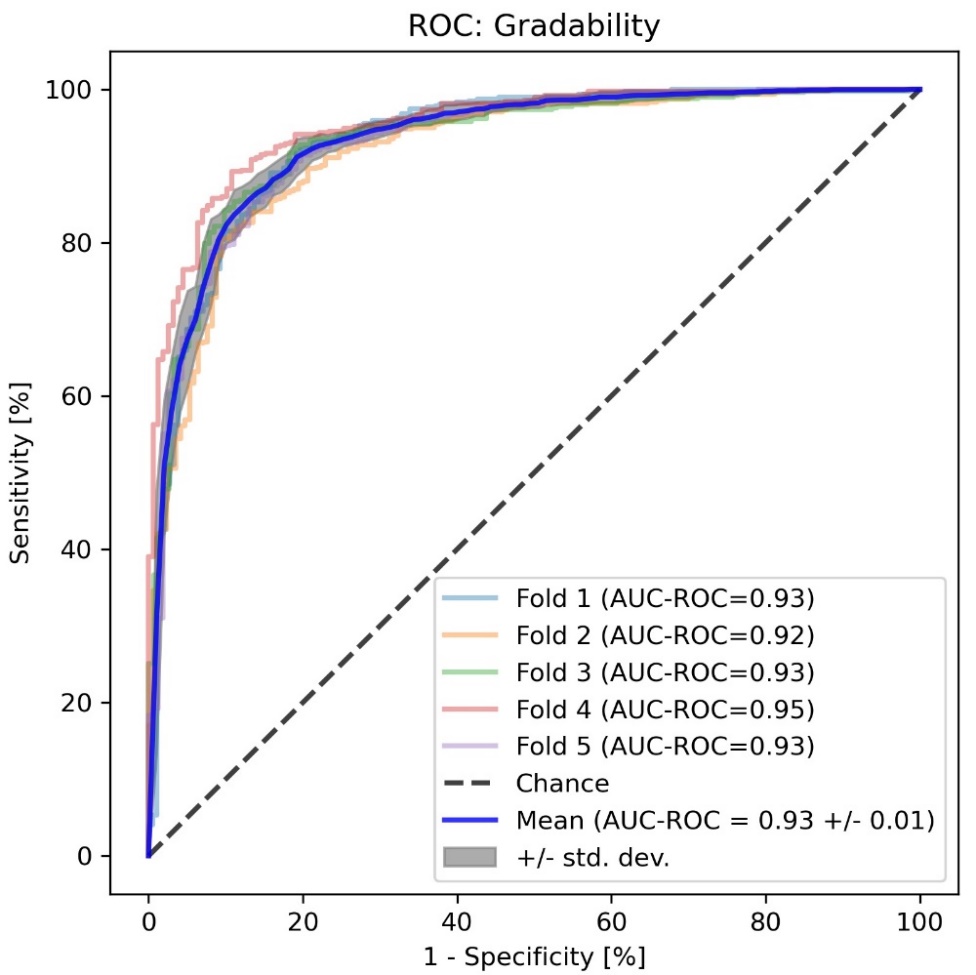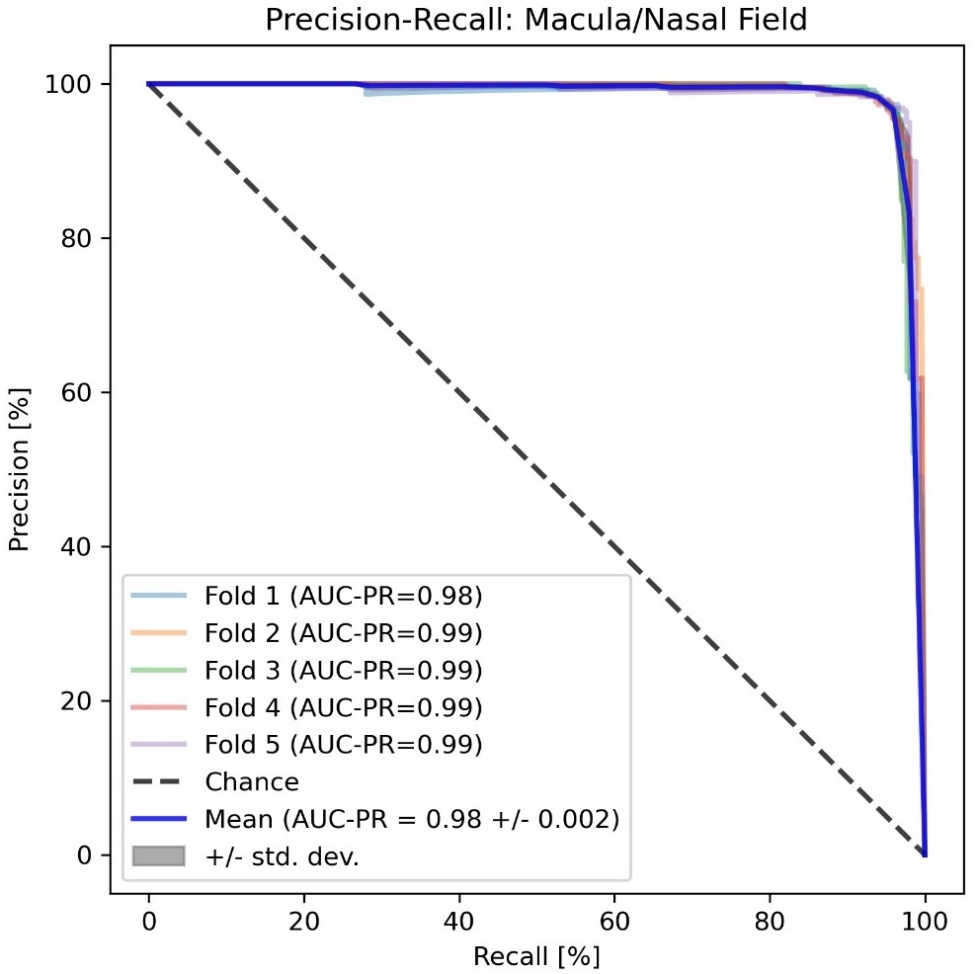 |

Figure S4**.** Data curation. Performance of curation models for gradeability scoring and field detection**.**

| 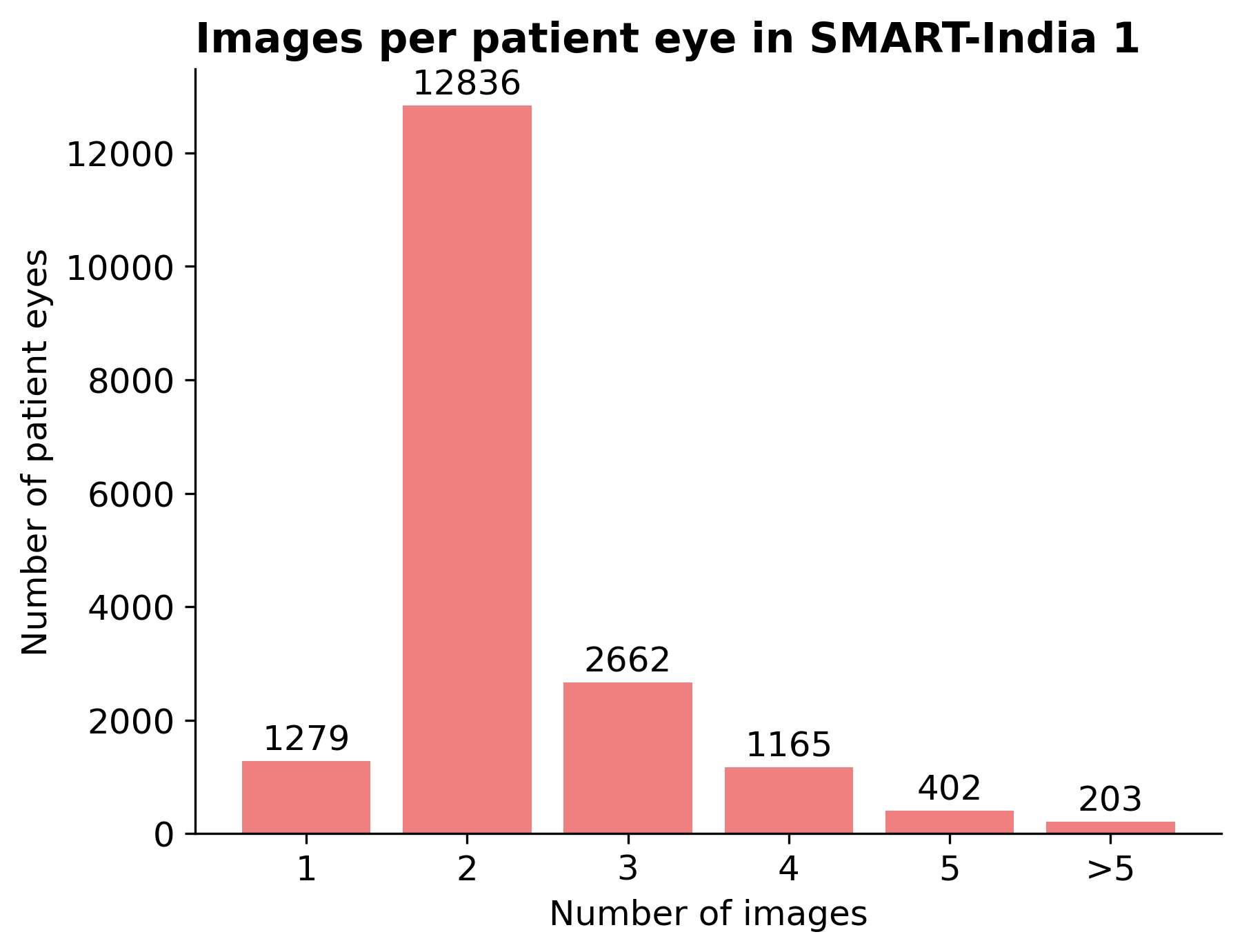 | 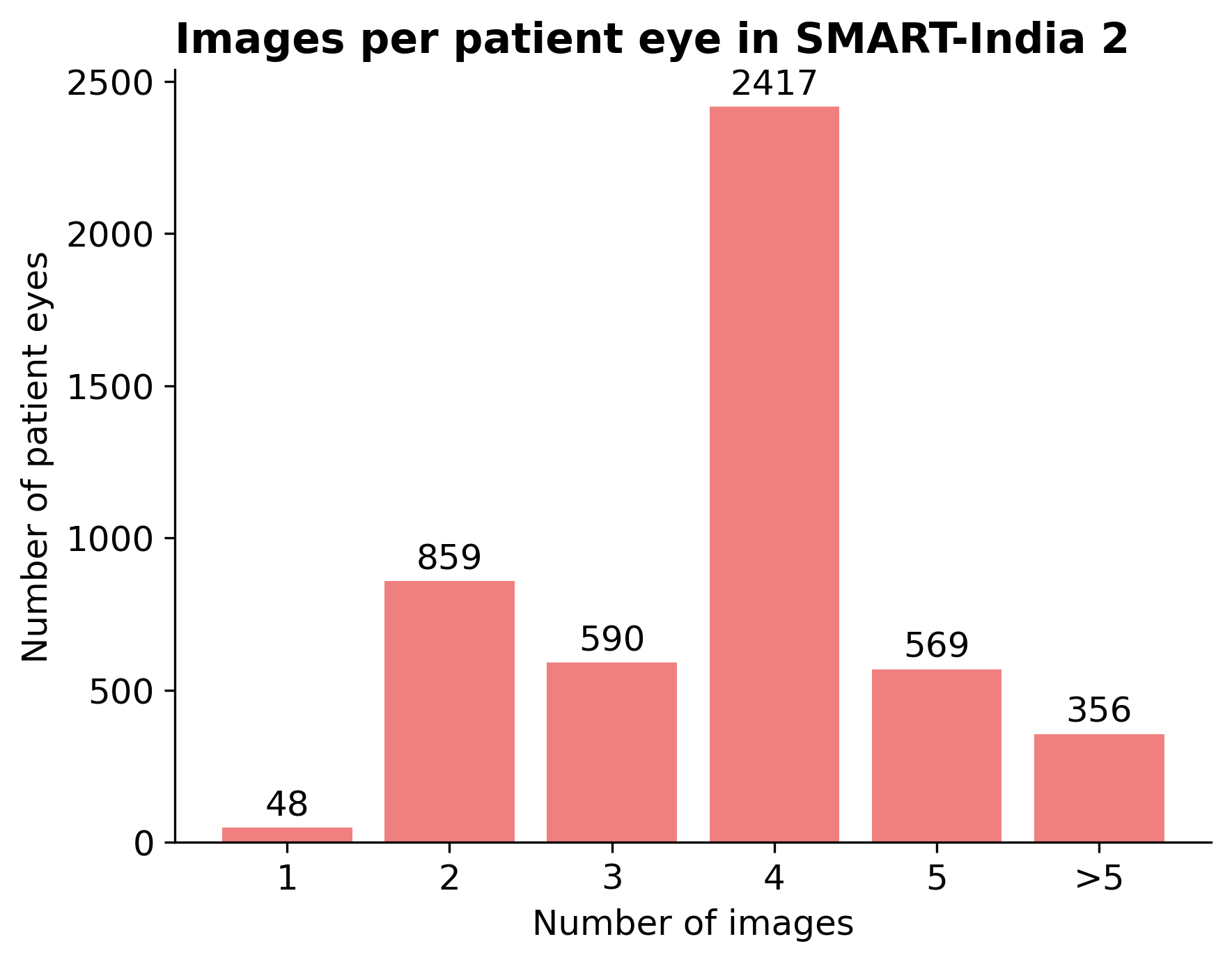 |
| --- | --- |

Figure S5**.** Images per patient eye. Distribution of the number of images per patient eye before 2-field patient eye dataset curation.

# Supplementary Tables

Table S1*.* Assessment of different fields and parts of the images. DLS performance achieved by each image region from the macula field and the optic disc field

| Macular field | AUROC | Optic disc field | AUROC |
| --- | --- | --- | --- |
| Full frame | 0.98 (0.97-0.98) | Full frame | 0.96 (0.95-0.98) |
| Optic disk | 0.85 (0.81-0.87) | Nasal | 0.91 (0.89-0.94) |
| Macula | 0.96 (0.95-0.96) | Optic disc | 0.89 (0.87-0.92) |
| Temporal | 0.92 (0.90-0.94) | Macula | 0.91 (0.90-0.94) |
| No optic disk | 0.97 (0.97-0.98) | No optic disc | 0.95 (0.95-0.97) |

Table S2*.* Predictive performance by risk factors.

|  | Number of Patients^1^ | AUROC |
| --- | --- | --- |
| BMI | 6,858 | 0.58 (0.54-0.63) |
| Duration of diabetes | 6,858 | 0.84 (0.81-0.86) |
| Systolic blood pressure | 6,858 | 0.59 (0.54-0.64) |
| Diastolic blood pressure | 6,858 | 0.49 (0.44-0.53) |
| Blood pressure  (systolic, diastolic) | 6,858 | 0.62 (0.57-0.67) |
| HbA_1c_ | 6,645 | 0.64 (0.59-0.67) |
| All risk factors | 4,645 | 0.84 (0.81-0.87) |
| DLS scores^2^ | 6,858 | 0.99 (0.98-0.99) |
| DLS | 6,858 | 0.99 (0.98-0.99) |

^1^Eyes with available data for the corresponding risk factors. ^2^Multivariate regression of DLS scores (both eyes)

Table S3**.** Training and evaluation data for the curation models.

| Fundus/non-fundus model | 3,514 images  *92.9% fundus, 7.1% non-fundus* |
| --- | --- |
| Laterality model | 3,261 fundus images  *48.3% left, 51.7% right* |
| Retinal field model | 3,245 fundus images  *60.5% macula-centred, 39.5% optic-disc-centred* |
| Gradeability model | 3,261 fundus images  *74.7% Gradable, 25.3% Ungradable* |

Table S4*.* Confusion matrix of the DLS for referable DR/DME stratified on DR annotations (True Positives and False Negatives are listed for each DR score, since False Positives and True Negatives can’t be defined in this analysis).

|  | | Ground truth^1^ | | | | |
| --- | --- | --- | --- | --- | --- | --- |
| Fold | DLS | No DR | Mild NPDR | Moderate NPDR | Severe NPDR | PDR |
| 0 | Non-referable | **2,316** | **119** | 42 | 0 | 5 |
|  | Referable | 56 | 41 | **197** | **26** | **113** |
| 1 | Non-referable | **2,297** | **113** | 41 | 0 | 5 |
|  | Referable | 60 | 33 | **159** | **30** | **105** |
| 2 | Non-referable | **2,254** | **102** | 33 | 0 | 2 |
|  | Referable | 79 | 40 | **185** | **37** | **86** |
| 3 | Non-referable | **2,312** | **99** | 44 | 1 | 3 |
|  | Referable | 49 | 23 | **195** | **27** | **94** |
| 4 | Non-referable | **2,272** | **109** | 28 | 0 | 1 |
|  | Referable | 71 | 29 | **163** | **17** | **100** |

^1^Patient eyes with Referable DME are excluded to evaluate performance exclusively on DR.

Table S5*.* Stratified results of the DLS for referable DR/DME on DR score, DME score and SM1/SM2 (True Positives and False Negatives are listed for each DR score, since False Positives and True Negatives can’t be defined in this analysis).

|  | | Ground truth^1^ | | |
| --- | --- | --- | --- | --- |
| Fold | DLS | No DME | Present DME | Referable DME |
| 0 | Non-referable | **2,429** | **6** | 0 |
|  | Referable | 97 | 0 | **4** |
| 1 | Non-referable | **2,410** | **0** | 1 |
|  | Referable | 90 | 3 | **5** |
| 2 | Non-referable | **2,349** | **7** | 4 |
|  | Referable | 116 | 3 | **4** |
| 3 | Non-referable | **2,408** | **3** | 2 |
|  | Referable | 71 | 1 | **5** |
| 4 | Non-referable | **2,380** | **1** | 1 |
|  | Referable | 100 | 0 | **9** |

^1^Patient eyes with Referable DR are excluded to evaluate performance exclusively on DME.

# Supplementary References

1. Nderitu, P. *et al.* Deep learning for gradability classification of handheld, non-mydriatic retinal images. *Sci. Rep.* **11**, 1–9 (2021).

2. Tan, M. & Le, Q. Efficientnet: Rethinking model scaling for convolutional neural networks. in *International conference on machine learning* 6105–6114 (PMLR, 2019).

3. Deng, J. *et al.* Imagenet: A large-scale hierarchical image database. in *2009 IEEE conference on computer vision and pattern recognition* 248–255 (Ieee, 2009).
